# DaBlaCa-16: Retrosigmoid Versus Conventional Ileal Conduit in Robot-assisted Radical Cystectomy, the MOSAIC Randomized Controlled Trial—Feasibility and 90-day Postoperative Complications

**DOI:** 10.1016/j.euros.2023.12.007

**Published:** 2024-01-04

**Authors:** Simone Buchardt Brandt, Stefanie Korsgaard Körner, Rikke Vilsbøll Milling, Ninna Kjær Nielsen, Pernille Skjold Kingo, Ulla Nordström Joensen, Lasse Bro, Thor Knak Jensen, Astrid Helene Livbjerg, Knud Fabrin, Marie-Louise Vrang, Michael Vangedal, Gitte Wrist Lam, Jørgen Bjerggaard Jensen

**Affiliations:** a Department of Urology, Aarhus University Hospital, Aarhus, Denmark; b Department of Clinical Medicine, Aarhus University, Aarhus, Denmark; c Department of Urology, Copenhagen University Hospital, Rigshospitalet, Copenhagen, Denmark; d Department of Clinical Medicine, Copenhagen University, Copenhagen, Denmark; e Department of Urology, Odense University Hospital, Odense, Denmark; f Department of Urology, Aalborg University Hospital, Aalborg, Denmark; g Department of Urology, Herlev and Gentofte University Hospital, Copenhagen, Denmark

**Keywords:** Ninety-day complications, Benign ureteroenteric strictures, Muscle-invasive bladder cancer, Radical cystectomy, Retrosigmoid ileal conduit, Robot-assisted surgery

## Abstract

**Background:**

Approximately 15% of patients undergoing radical cystectomy (RC) develop benign ureteroenteric strictures. Of these strictures, the majority are located in the left ureter. To lower the rate of strictures, a retrosigmoid ileal conduit has been suggested.

**Objective:**

To investigate the feasibility and safety of a retrosigmoid ileal conduit during robot-assisted RC in bladder cancer patients.

**Design, setting, and participants:**

This randomized controlled trial included 303 patients from all five cystectomy centers in Denmark from May 2020 to August 2022. Participants were diagnosed with bladder cancer and scheduled for robot-assisted RC with an ileal conduit.

**Intervention:**

Intervention group: a retrosigmoid ileal conduit was constructed using approximately 25 cm of the terminal ileum and tunneled behind the sigmoid where the left ureter was anastomosed from end to side. Control group: the conventional ileal conduit ad modum Bricker with individual end-to-side anastomoses.

**Outcome measurements and statistical analysis:**

Patients were analyzed by the intention-to-treat approach. Complications within 90 d were categorized using the Clavien-Dindo grading system and compared using Fisher’s exact test. Wilcoxon’s test was used for pre- and postoperative renal function.

**Results and limitations:**

Of the 149 patients randomized for the retrosigmoid ileal conduit (MOSAIC), a total of 137 (92%) patients received the allocated conduit. Postoperative complications were distributed equally between the two groups. The relative risk of Clavien-Dindo complications of grade ≥III was 1.12 (95% confidence interval: 0.96–1.31) in the intervention group compared with the control group.

**Conclusions:**

The retrosigmoid ileal conduit with robot-assisted RC was technically feasible. Early postoperative complications were not significantly different when comparing the two groups. Further investigation of long-term complications, including strictures, is needed.

**Patient summary:**

We compared a conventional urinary diversion with a longer conduit to prevent constriction from developing in the ureters. The new conduit is feasible and safe within the first 90 d, with no differences in postoperative complications from those of the conventional diversion.

## Take Home Message

The MOSAIC study is a multicenter randomized controlled trial investigating a retrosigmoid ileal conduit. The study randomized 303 patients between the conventional and retrosigmoid conduits, and found that the retrosigmoid ileal conduit is feasible and safe within 90 d.

## 1 Introduction

Radical cystectomy (RC) with a urinary diversion is the gold standard when treating muscle-invasive bladder cancer [1]. RC is associated with several complications—gastrointestinal, infectious, wound related, cardiac, and genitourinary including strictures [2]. Benign ureteroenteric strictures are diagnosed in 12–20% of cystectomized patients and are usually diagnosed within the first 2 yr postoperatively [3], [4], [5].

**Fig. 1:**
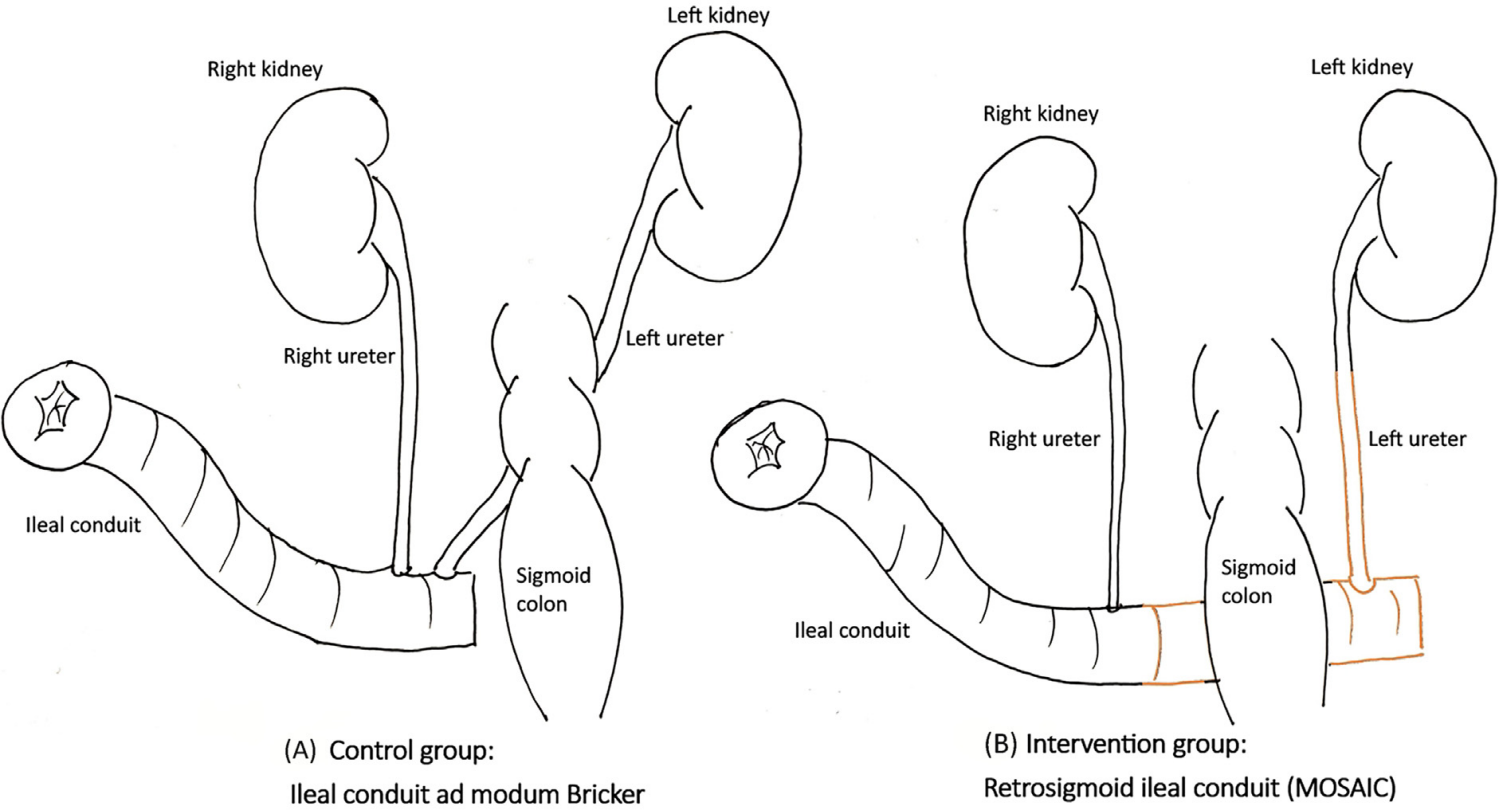
Illustration of the conduits and their different placements in relation to the sigmoid colon: (A) the conventional ileal conduit ad modum Bricker and (B) the retrosigmoid ileal conduit (MOSAIC).

The majority of strictures occur in the left ureter [6], [7]. Presumably due to the typical ileal conduit with retrosigmoid transposition of the left ureter, leaving the left ureter longer and therefore more vulnerable to ischemia due to compromised vascular supply (Fig. 1A).

Previously, a few small nonrandomized studies have investigated a retrosigmoid ileal conduit with the intent to lower the risk of strictures [8], [9], [10]. The ileal segment used for the retrosigmoid conduit is longer than the conventional conduit. Thereby, the presumed more robust ileal segment will cross under the mesentery instead of the left ureter (Fig. 1B). Thus, the left ureter is divided more proximally, needs less mobilization, and avoids compression from the colon. This will potentially lower the risk of ischemia and thereby lower the risk of stricture.

Li et al. [8] were first to investigate the retrosigmoid ileal conduit. With 42 patients undergoing the modified technique, they found no increased risk of perioperative, or early and intermediate complications in relation to the conduit [8]. In 2019, Ficarra et al. [9] found that the number of diagnosed strictures was significantly lower in the group with the retrosigmoid conduit and without an increased risk of complications than in the conventional ileal conduit group. However, this was a nonrandomized, small cohort of 67 patients and the follow-up period was significantly longer in the control group. Moreover, all previous series published on retrosigmoid conduit are open surgery series, whereas the robotic approach has not been investigated previously.

Robot-assisted radical cystectomy (RARC) has been introduced as a minimally invasive technique over the past decades, and has been proved to be safe and at least equivalent to open surgery regarding surgical outcomes [11].

The primary aim of the MOSAIC trial is to investigate the number of left-sided strictures. MOSAIC (Randomized Controlled Trial with a Modified Urinary Conduit to Lower Strictures After Radical Cystectomy) is a clinical trial. In the present paper, we aim to report the outcome regarding the feasibility of RARC with an intracorporeal retrosigmoid ileal conduit in bladder cancer patients. Moreover, we compare the safety regarding postoperative complications and renal function within 90 d when comparing the conventional ileal conduit ad modum Bricker with the retrosigmoid ileal conduit in a randomized controlled trial.

## 2 Patients and methods

### 2.1 Study design

This open-label, randomized controlled trial was conducted from May 2020 until August 2022. In total, 303 patients were enrolled from all five centers performing RC in Denmark. The protocol was approved by the Central Denmark Region Committee on Health Research Ethics (1-10-72-72-20), and was registered on clinicaltrials.gov (NCT04391790) and on the internal list of research projects in the Central Denmark Region approved by the Danish Data Protection Agency. Informed written consent was obtained before randomization.

### 2.2 Eligibility criteria

Patients eligible for inclusion were diagnosed with bladder cancer and scheduled for RARC, with or without neoadjuvant chemotherapy, and planned for an ileal conduit.

The exclusion criteria were previous abdominal or pelvic radiotherapy, previous major abdominal surgery involving resection of the bowel or construction of an enteric stoma, planned left-sided urostomy, complete ureteral duplication, and solitary or single-functioning kidney.

Randomization between the intervention and control groups was performed 1:1 using REDCap software (REDCap, Nashville, TN, USA), hosted at Aarhus University (Aarhus, Denmark) [12]. An external data manager created the algorithm for randomization. Patients were stratified according to the presence of hydronephrosis with hydroureter and the cystectomy center.

### 2.3 Surgical technique

Standard RARC with extended lymph node dissection was performed in both groups by a surgeon experienced in RARC with the intracorporeal conventional ileal conduit ad modum Bricker. Both ureters were divided approximately at the crossing of the medial umbilical ligament.

After completing RC and extended lymph node dissection, the surgeon identified the ileal segment for the ileal conduit, with the anal end approximately 20–25 cm from the ileocecal junction. The division was routinely made with a stapling device. Continuity of the intestine was re-established using a stapling device.

The resection of the right ureter was approximately at the crossing of the iliac vessels in both study groups. The ureter was spatulated, stented, and anastomosed using an individual end-to-side technique with monofilament 4-0 absorbable running sutures.

### 2.4 Control group—intracorporeal ileal conduit ad modum Bricker

An ileal segment of approximately 15 cm was used. The incisions for the ureter anastomoses were made approximately 2 cm apart in the oral end of the left incision placed approximately 1 cm from the stapling line.

The left ureter was mobilized gently at least 5 cm above its crossing of the left iliac artery, and then mobilized to the right side of the abdomen through a wide retrosigmoid passage. The ureter was spatulated at the distal end where the surgeon ensured a relevant length to achieve an anastomosis without inappropriate stretching of the tissue. After spatulation, the ureter was stented and anastomosed using an end-to-side technique with monofilament 4-0 absorbable running suture.

### 2.5 Intervention group—intracorporeal retrosigmoid ileal conduit (MOSAIC)

An ileal segment of approximately 25 cm was used. The two incisions for the ureter anastomoses in the ileal segment were made with approximately 8–10 cm distance to each other in the oral end, with the left incision placed approximately 1 cm from the stapling line. The ileal segment’s oral end was then tunneled retrosigmoidally to the left side of the abdomen where it could be attached to the psoas muscle tendon, to provide extra retraction to do the anastomosis.

The left ureter was resected at the crossing of the iliac vessels. It was spatulated, stented, and anastomosed using an end-to-side technique with monofilament 4-0 absorbable running sutures. After completing the anastomosis, the potential psoas attachment was removed based on the surgeon’s discretion.

### 2.6 Data registration and outcomes

Staging of bladder cancer was categorized from the transurethral resection of bladder tumor (TURBT) as muscle-invasive or non–muscle-invasive bladder cancer and by examination of the cystectomy specimen as organ confined (≤T2N0) or non–organ confined (>T2 or N+).

Evaluation of short-term renal function was performed using creatinine test and renography. Creatinine was measured preoperatively and at every visit in the outpatient clinic after surgery. Creatinine clearance was calculated using the Cockcroft-Gault equation with adjusted body weight when body mass index (BMI) was ≥25 [13]. Renography was performed pre- and postoperatively to assess distribution of renal function.

Perioperative clinical data were collected continually. Complications were registered and classified according to the Clavien-Dindo grading system within 90 d by a single unblinded investigator.

### 2.7 Power calculation

Previous studies have reported stricture incidences of approximately 15% after RARC [14], [15]. The primary endpoint in the MOSAIC study is left-sided strictures within 2 yr. We assumed that 15% of patients in the control group would develop left-sided strictures compared with 5% in the intervention group. With 5% alpha and 80% power in a two-sided test, 140 patients were required in each group according to the primary endpoint. Expecting a dropout of ten patients in both groups after randomization, we aimed to enroll 300 patients.

### 2.8 Statistical analysis

A statistical analysis was performed using R version 4.2.2 (Boston, MA, USA) [16]. All statistics were performed as an intention-to-treat analysis. Continuous variables were compared between the two groups using Wilcoxon rank sum test and summarized with standard descriptive statistics, including median and interquartile range. Categorical variables were summarized with frequencies and percentages, and were compared using Fisher's exact test. Relative risk (RR) with a 95% confidence interval (95% CI) was estimated to compare the risk of Clavien-Dindo complications of grade ≥III in the intervention group compared with the control group.

## 3 Results

**Fig. 2:**
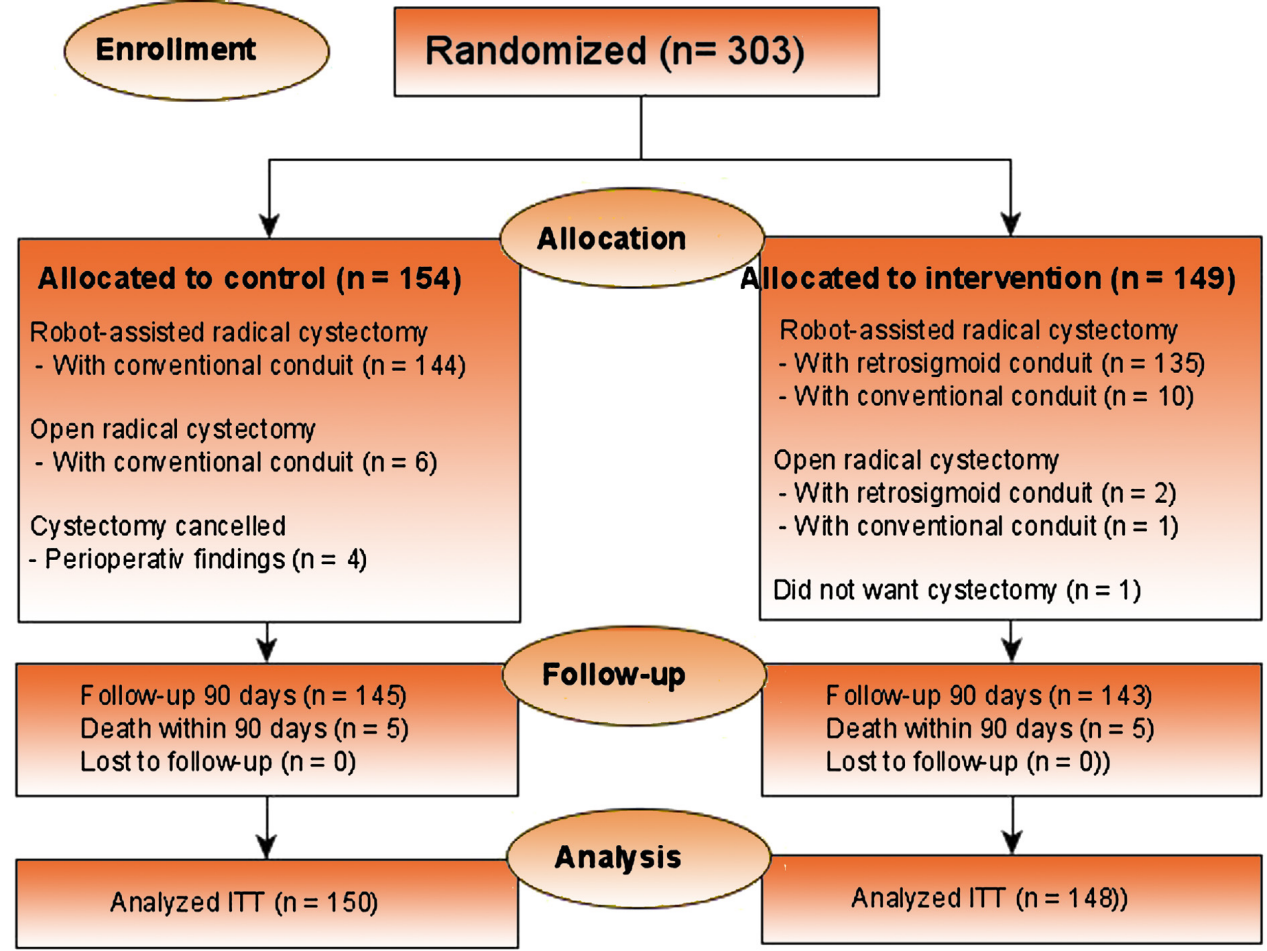
CONSORT 2010 diagram. CONSORT = Consolidated Standards of Reporting Trials; ITT = intention to treat.

In total, 303 patients were enrolled in the MOSAIC study from May 2020 to August 2022. Table 1 shows the demographics of the study population at baseline. The CONSORT diagram in Figure 2 shows information regarding enrollment, allocation of intervention, and follow-up. After inclusion and randomization, five patients did not undergo cystectomy. In four out of the five patients, cystectomy was canceled due to an assessment of perioperative findings (two patients with advanced stages of bladder cancer and one patient with severe adhesions, and one patient could not tolerate the anesthesia with a severe drop in blood pressure), and one patient chose a second TURBT instead. Of the 149 patients randomized to the intervention group, 135 received the allocated RARC with an intracorporeal retrosigmoid ileal conduit (MOSAIC) and two patients received open RC. The conventional conduit was chosen intraoperatively in ten patients due to the following: forgetfulness of the involved surgeon or misconception of the protocol (five patients), large or fatty sigmoid colon (three), inability to identify the cecum (one patient), or adhesions (one patient).

**Table 1:** Characteristics of the study population

Characteristics	Conventional ileal conduit	Retrosigmoid ileal conduit
	(*n* = 150)	(*n* = 148)
Age (yr), median (IQR)	72 (66, 76)	72 (66, 76)
Male, *n* (%)	119 (79)	117 (79)
BMI (kg/m^2^), median (IQR)	26 (24, 29)	27 (24, 29)
T stage at TURBT, *n* (%)		
NMIBC	54 (36)	61 (41)
MIBC	96 (64)	87 (59)
Staging at cystectomy, *n* (%)		
Organ confined	114 (77)	98 (67)
Non-organ confined	35 (23)	49 (33)
Neoadjuvant chemotherapy, *n* (%)	55 (37)	54 (38)
Smoking status, *n* (%)		
Never	27 (18)	30 (20)
Former ≤5 yr ago	19 (13)	16 (11)
Former >5 yr ago	66 (44)	68 (46)
Smoker	33 (22)	31 (21)
NA	4 (2.7)	2 (1.4)
Age-adjusted Charlson comorbidity index score, *n* (%)		
CCI <3	12 (8.0)	11 (7.4)
CCI ≥3	138 (92)	137 (93)
ASA physical status classification, *n* (%)		
I	8 (5.4)	7 (4.8)
II	99 (67)	92 (63)
III	40 (27)	46 (32)
Preoperative hydronephrosis with hydroureter, *n* (%)		
Yes	21 (14)	19 (13)
No	129 (86)	129 (87)
Operating center, *n* (%)		
Aarhus University Hospital	53 (35)	54 (36)
Herlev and Gentofte University Hospital	33 (22)	33 (22)
Aalborg University Hospital	26 (17)	28 (19)
Odense University Hospital	23 (15)	19 (13)
Copenhagen University hospital Rigshospitalet	15 (10)	14 (9.5)

ASA = American Society of Anesthesiologists; CCI = Charlson comorbidity index; IQR = interquartile range; MIBC = muscle-invasive bladder cancer; NA = not available; NMIBC = non–muscle-invasive bladder cancer; TURBT = transurethral resection of bladder tumor.

In total, nine patients were converted to open RC, of whom three were in the intervention group. Two of these three patients were converted to open RC with the allocated retrosigmoid ileal conduit (MOSAIC) because of no available RARC-approved surgeon on the day of surgery. The last patient was converted to open RC due to difficulties identifying the cecum, and the patient received a conventional ileal conduit. The reasons for converting the six patients in the control group were difficulties to anesthetize two patients, a lack of an RARC-approved surgeon on the day of surgery for two patients, advanced disease for one patient, and sever adhesions for the last patient.

No patients have been diagnosed with a ureteroenteric stricture within the first 90 d postoperatively.

### 3.1 Surgery

Table 2 shows a comparison of the two groups’ surgical characteristics. No significant difference was found when comparing operative time, length of stay, or days until bowel movement. The resected part of the left ureter was significantly longer in the intervention group than in the control group (5.50 vs 3.90 cm; *p* < 0.001). When comparing the length of the resected right ureter in the two groups, we found no significant difference (*p* = 0.60). A psoas hitch was carried out in 20/148 (13.5%) patients in the intervention group, and the psoas hitch was removed in 13/20 (65%) patients. A total of 31 patients in the intervention group had a BMI of >30 kg/m^2^. Two of these 31 (6.5%) surgeries were converted to a conventional ileal conduit due to anatomical difficulties.

**Table 2:** Perioperative characteristics of the radical cystectomy and the hospitalization afterward

	Conventional ileal conduit	Retrosigmoid ileal conduit	*p* value
	(*n* = 150)	(*n* = 148)	
Operation, *n* (%)			0.50
Robot-assisted RC	144 (96)	145 (98)	
Open RC	6 (4.0)	3 (2.0)	
Operative time (min), median (IQR)	288 (248, 362)	298 (239, 366)	0.73
Blood loos (ml), median (IQR)	150 (100, 300)	150 (50, 250)	0.47
Resected ureters (cm), median (IQR)			
Left ureter	3.90 (2.50, 5.00)	5.50 (4.00, 7.00)	<0.001
Right ureter	5.00 (3.77, 6.50)	5.00 (3.50, 6.03)	0.60
Length of stay (d), median (IQR)	7 (5, 10)	8 (6, 13)	0.081
Days until flatus, median (IQR)	2 (2, 3)	3 (2, 3)	0.46
Days until bowel movement, median (IQR)	4 (3, 5)	4 (3, 5)	0.23

IQR = interquartile range; RC = radical cystectomy.

### 3.2 Surgical complications

Distribution of the Clavien-Dindo grades of the 90-d complications was equal in the two groups (Table 3). Major complications (Clavien-Dindo grade ≥III) were observed in 52/148 (35%) patients in the intervention group and in 41/150 (27%) patients in the control group. The RR of the major complications was 1.12 (95% CI 0.96; 1.31) in the intervention group compared with the control group (*p* = 0.17).

**Table 3:** Postoperative 90-d complications, including complications classified according to the Clavien-Dindo grading system

	Conventional ileal conduit	Retrosigmoid ileal conduit	*p* value
	(*n* = 150)	(*n* = 148)	
Anastomotic leak from ureter, *n* (%)	11 (7.3)	13 (8.8)	0.65
Anastomotic leak from bowel, *n* (%)	1 (0.7)	0 (0)	>0.99
Urosepsis, *n* (%)	14 (9.3)	17 (11)	0.54
Pyelonephritis, *n* (%)	7 (4.7)	7 (4.7)	0.98
Nephrostomy, *n* (%)	11 (7.3)	17 (11)	0.22
Mechanical ileus, *n* (%)	6 (4.0)	9 (6.1)	0.41
Highest Clavien-Dindo grade, *n* (%)			0.32
No complications	40 (27)	26 (18)	
I	24 (16)	20 (14)	
II	45 (30)	50 (34)	
IIIa	12 (8.0)	19 (13)	
IIIb	19 (13)	24 (16)	
IVa	3 (2.0)	5 (3.4)	
IVb	2 (1.3)	0 (0)	
V	5 (3.3)	4 (2.7)	
Clavien-Dindo grade ≥III, *n* (%)	41 (27)	52 (35)	0.15

### 3.3 Renal function

Table 4 shows the surgical impact on early renal function. Creatinine was measured preoperatively and a median of 114 d (100; 124) postoperatively. The postoperative renography was performed after a median of 107 d (71; 118). The ratio of individual preoperative left renal function to postoperative renal function on renography was 1.00 (0.95; 1.05) in the control group and 1.00 (0.94; 1.07) in the intervention group (*p* = 0.80).

**Table 4:** Comparison of short-term renal function

	Conventional ileal conduit	Retrosigmoid ileal conduit	*p* value
	(*n* = 150)	(*n* = 148)	
Creatinine			
Preoperative (μmol/l), median (IQR)	79 (68, 94)	81 (70, 99)	0.48
Postoperative (μmol/l), median (IQR)	83 (71, 97)	87 (76, 104)	0.25
Patient individual ratio preoperative/postoperative creatinine, median (IQR)	0.95 (0.85, 1.05)	0.95 (0.85, 1.06)	0.96
CrCl—preoperative (ml/min), median (IQR)	76 (61, 92)	72 (58, 93)	0.26
CrCl—postoperative (ml/min), median (IQR)	72 (57, 87)	68 (58, 88)	0.75
Renography (%), median (IQR)			
Left renal function—preoperative	50 (47, 54)	50 (47, 55)	0.77
Left renal function—postoperative	50 (47, 54)	50 (47, 55)	0.46

CrCl = creatinine clearance (Cockcroft-Gault equation); IQR = interquartile range.

## 4 Discussion

Strictures are hypothesized to originate largely from distal ischemia, thereby explaining the large portion of left-sided strictures. The growing interest in the left ureter and the level of resection in RC suggest a need for a new ileal conduit [8], [9], [10], [17].

This is the first study to investigate the retrosigmoid ileal conduit by an intracorporeal technique and the first randomized study that compares the retrosigmoid conduit with the conventional ileal conduit. We found that RARC with a retrosigmoid ileal conduit (MOSAIC) was feasible and safe within the immediate postoperative period. Thus, only nine out of 298 (3%) patients were converted to open RC, of whom only three were in the intervention group, indicating the feasibility of the intracorporeal retrosigmoid ileal conduit (MOSAIC) surgery.

Li et al. [8] first published a retrospective paper on 42 patients undergoing open RC with the retrosigmoid ileal conduit. Ficarra et al. [9] performed the first observational study with both the control and the study group with open RC. In this study, the retrosigmoid ileal conduit was performed in 30 patients compared with 37 patients receiving the conventional ileal conduit, with ureteric implantation made with the Wallace technique.

In the present study, one of the secondary outcomes was to investigate the safety of the procedure by examining immediate postoperative complications. We found that 90-d complications were equally distributed in the two groups. Ficarra et al. [9] observed a low rate of major complications (Clavien-Dindo grade ≥III) in 15/67 patients (22.38%). This was not significantly different between the control and intervention groups, which is in good agreement with the results of our study, where the retrosigmoid ileal conduit (MOSAIC) had a risk of major complications, which was not significantly different from the conventional ileal conduit. These findings were obtained despite the well-known learning curve of new procedures [18]. None of the surgeons were experienced in performing the retrosigmoid ileal conduit; however, all were experienced in the conventional ileal conduit ad modum Bricker or they were supervised by an experienced surgeon.

The negative impact of RC on kidney function is known but has not previously been evaluated with the retrosigmoid ileal conduit [19]. In our study, evaluation of short-term renal function after RC with serum creatinine and renography shows no differences between the two conduits. However, the methods for evaluating renal function could be inadequate. The creatinine fluctuates, and the renography is a relative measure between the two units. However, the results indicate equal renal function in both groups within this immediate postoperative period.

A limitation of the present study is the detailed knowledge of reproducibility in a clinical everyday setting as we did not make a detailed screening log during the study period. Nevertheless, the baseline information of the MOSAIC study patients is comparable with that of other Danish cohorts describing patients undergoing cystectomy [20]. The reporting of complications could be limited by the open-label design and the lack of prospective registration of intraoperative complications by the surgeon, according to the European Association of Urology guidelines [21]. However, the grading of 90-d complications was distributed uniformly across the study groups.

BMI is also a possible limitation. Patient BMI values were a median of 26 and 27 kg/m^2^, with the highest being 45 kg/m^2^ in the intervention group. Tunneling of the conduit could potentially be more difficult when BMI increases; however, we did not find the feasibility to be dependent on the patients’ BMI.

In the present study, patients previously treated with radiotherapy in the pelvic area were excluded, thereby limiting the documented feasibility to patients without previous treatment with radiotherapy. This limitation is important as the risk of strictures has been shown to be higher in this patient group in previous studies [22].

The present study makes a noteworthy contribution to the knowledge of the optimal ileal conduit. Hopefully, the technique will lower the rate of left-sided strictures in the longer term, as indicated by previous studies. Moreover, when the left ureter, for other reasons, is resected more extensively, the RARC with intracorporeal retrosigmoid ileal conduit (MOSAIC) has been proved to be both a feasible and a safe approach.

An assessment of the effect of the retrosigmoid ileal conduit on left-sided strictures with a longer follow-up is needed.

## 5 Conclusions

This study shows that RARC with a retrosigmoid ileal conduit (MOSAIC) is feasible and safe regarding 90-d follow-up compared with RARC with a conventional ileal conduit. Long-term follow-up results concerning strictures and metabolic complications are pending.

  ***Author contributions:*** Simone Buchardt Brandt had full access to all the data in the study and takes responsibility for the integrity of the data and the accuracy of the data analysis.

  *Study concept and design*: J.B. Jensen, Brandt, Vangedal, Lam.

*Acquisition of data*: Brandt, Joensen, Bro, T.K. Jensen, Livbjerg, Fabrin, Vrang, Vangedal, Lam.

*Analysis and interpretation of data*: J.B. Jensen, Brandt, Kingo, Körner, Milling, Nielsen.

*Drafting of the manuscript*: Brandt, J.B. Jensen, Kingo.

*Critical revision of the manuscript for important intellectual content*: Körner, Milling, Nielsen, Joensen, Bro, T.K. Jensen, Livbjerg, Fabrin, Vrang, Vangedal, Lam.

*Statistical analysis*: Brandt.

*Obtaining funding*: J.B. Jensen, Brandt.

*Administrative, technical, or material support*: Brandt.

*Supervision*: J.B. Jensen, Lam, Kingo.

*Other*: Inclusion of patients and surgery: Brandt, Körner, Milling, Nielsen, T.K. Jensen, Kingo, Joensen, Bro, J.B. Jensen, Livbjerg, Fabrin, Vrang, Vangedal, Lam.

  ***Financial disclosures:*** Simone Buchardt Brandt certifies that all conflicts of interest, including specific financial interests and relationships and affiliations relevant to the subject matter or materials discussed in the manuscript (eg, employment/affiliation, grants or funding, consultancies, honoraria, stock ownership or options, expert testimony, royalties, or patents filed, received, or pending), are the following: None.

  ***Funding/Support and role of the sponsor:*** The study was supported by Christian Larsen and Judge Ellen Larsens Fond, and Louis-Hansens Fond.

  ***Acknowledgments:*** We thank research nurses from all five sites. We would also like to extend our gratitude to the participating surgeons and surgical staff in the trial.
